# Metabolomic profiles in yak mammary gland tissue during the lactation cycle

**DOI:** 10.1371/journal.pone.0219220

**Published:** 2019-07-05

**Authors:** Zhixiong Li, Mingfeng Jiang

**Affiliations:** 1 College of Life Science and Technology, Southwest Minzu University, Chengdu, Sichuan, China; 2 Sichuan Provincial Key Laboratory of Qinghai-Tibetan Plateau Animal Genetic Resource Conservation and Exploitation, Chengdu, Sichuan, China; University of Illinois, UNITED STATES

## Abstract

The yak is one of the most important domestic animals in Tibetan life for providing basic resources such as milk, meat and transportation. Although yak milk production is not elevated, yak milk is superior to dairy cow milk in nutrient composition (protein and fat). However, the understanding of the metabolic mechanisms of yak mammary gland tissue during the lactation cycle remains elusive. In this study, GC-MS-based metabolomics was employed to study the metabolic variations in the yak mammary gland during the lactation cycle (pregnancy, lactation and dry period). Twenty-nine metabolites were up or downregulated during the lactation period. Compared to the dry period, during the lactation period the levels of oxalic acid were upregulated, while glycine and uridine were downregulated. Thirty-seven pathways were obtained when the 29 differential metabolites were imported into the KEGG pathway analysis. The most impacted pathways during the lactation cycle were glycine, serine and threonine metabolism; alanine, aspartate and glutamate metabolism; TCA cycle; glyoxylate and dicarboxylate metabolism; and pyrimidine metabolism. Our results provide important insights into the metabolic events involved in yak mammary gland development, lactogenesis and lactation, which can guide further research to improve milk yield and enhance the constituents of yak milk.

## Introduction

The yak (*Bos grunniens*) lives in regions centered on the Chinese Qinghai-Tibet Plateau, including the highlands of Nepalese Himalayas, Indian Kasmir, Tibet, Mongolia and Bhutan [1] at high altitudes from 2000–5000 m where few other animals can survive. The number of yaks that live in China is approximately 13 million, comprising 92.8% of the gross number of yaks on this planet [2]. Yaks can use the pasture resources of this area and provide transportation, leather, meat and milk. Yak milk and milk products are the major dietary ingredients for 6.5 million Tibetan people as well as it is an important source of income to the local families. For these reasons, the yak is one of the most important domestic animals in Tibetan life [3], and the yak daily industry has rapidly grown in recent years [4].

Although yaks have a limited production of milk (~3 kg/day), their milk is substantially richer in nutrients than dairy cows [5]. Yak milk contains 16.9%-17.7% solids, including 4.9%-5.3% protein, 5.5%-7.2% fat, 4.5%-5.0% lactose, and 0.8%-0.9% minerals [5]. A better understanding of the inherent metabolic status during different stages of lactation in yak mammary gland can enlighten important metabolic processes, which determines milk production and composition.

Metabolomics can be a powerful approach to investigate the low-molecular-weight metabolites and provide a global understanding of physiological alterations in specific organs/tissues [6]. In a previous study, the metabolite profile of yak milk was evaluated [7]; however, there is a lack of information regarding the metabolic profile of yak mammary gland. Considering the different physiological alterations of mammary gland during the lactation cycle, we hypothesized that the metabolite profile of yak mammary gland will also change over the lactation stage. The objective of the present study was to investigate the metabolite profile of yak mammary gland tissue during pregnancy, lactation and dry period by gas chromatography/mass spectrometry (GC-MS).

## Materials and methods

### Ethics statement

The animal care and use were performed according to the regulations for the Administration of Affairs Concerning Experimental Animals (Ministry of Science and Technology, China, revised in June 2004) and approved by the Institutional Animal Care and Use Committee, Southwest Minzu University, Chengdu, Sichuan, China.

### Yaks and sample collection

The surgical procedure for yak mammary biopsy was performed in accordance with the operations guide to ameliorate animal suffering. Seven healthy Maiwa yaks (4 years old) with unrelated background were obtained from Longri Breeding Farm of Sichuan Province, Hongyuan County, Sichuan Province, China. Individuals examined from pregnancy to the dry period were divided in to nine subgroups, class T1 (n = 5, -30±2 d), class T2 (n = 6, -15±2d), class T3 (n = 6, +1 d), class T4 (n = 7, +15 d), class T5 (n = 7, +30 d), class T6 (n = 6, +60 d), class T7 (n = 7, +120 d), class T8 (n = 7, +180 d), and class T9 (n = 6, +240 d) according to their parturition (d). Class T1 and T2 belonged to the pregnancy period, Class T3 to T8 belonged to the lactation period, Class T9 belonged to the dry period. A total of 57 mammary gland tissue samples were collected in the present study. Mammary gland tissue samples (approximately 500 mg) were collected by biopsy of the right or left rear quarters as previously described [8]. All samples were immediately frozen and stored in liquid nitrogen.

### Metabolite profiling analysis

Mammary gland tissue samples (~100 mg) was ground and transferred to 2-mL centrifuge tubes. A thousand μL of 80% methanol (pre-cooled at -20°C) was then added and vortexed for 30 s, followed by the addition of 60 μL of 2-Chloro-L-phenylalanine (0.2 mg/mL stock in methanol) and 60 μL of heptadecanoic acid (0.2 mg/mL stock in methanol) as internal quantitative standards; the mixture was vortexed for 30 s. The tubes were placed into an ultrasound machine at room temperature for 30 min, incubated on the ice for 30 min, and centrifuged at 14,000 rpm at 4°C for 10 min; subsequently, 0.8 mL of supernatant was transferred to a new centrifuge tube. The samples were blow-dried by moderate nitrogen, and 60 μL of 15 mg/mL methoxyamine pyridine solution was then added, followed by vortexing for 30 s and incubation for 120 min at 37°C. Sixty μL of BSTFA reagent (containing 1% TMCS) was added to the mixture, followed by incubation for 90 min at 37°C. Finally, the samples were examined for the metabolite contents by using GC-MS. A quality control (QC) sample was also prepared by mixing equal volumes of each sample; the samples were aliquoted for analysis prior to sample preparation. The QC samples were used to monitor deviations of the analytical results from the pooled mixtures and compare to the errors caused by the analytical instrument itself.

GC-MS data were acquired using Agilent 7890A GC system coupled to an Agilent 5975C inert XL EI/CI mass spectrometric detector (MSD) system (Agilent Technologies, Santa Clara, CA, USA). GC was performed on an HP-5MS capillary column (5% phenyl/95% methyl polysiloxane [30 m × 250 μm i.d., 0.25 μm film thickness, Agilent J & W Scientific, Folsom, CA, USA]) to separate the derivatives at a constant flow of 1 mL/min helium. A 1-μL aliquot of the sample was injected in split mode in a 20:1 split ratio by the auto-sampler. The injection temperature was 280°C, the interface was set to 150°C and the ion source was adjusted to 230°C. The temperature-rise programs included an initial temperature of 80°C for 5 min, followed by a 20°C/min increased up to 300°C and a hold at 300°C for 6 min. Mass spectrometry was determined by the full-scan method with a range from 35 to 500 m/z.

### Data analysis

Raw GC-MS data were converted into CDF format (NetCDF) files by Agilent GC/MS 5975 Data Analysis software and subsequently processed by XCMS (www.bioconductor.org) [9, 10]. The signal integration area of each metabolite was normalized with the total peak area for each sample. The identification of metabolites using the Automatic Mass Spectral Deconvolution and Identification System (AMIDS) was searched against commercially available databases such as the National Institute of Standards and Technology (NIST) and Wiley libraries. Metabolites were confirmed by comparison of mass spectra and retention indices to the spectra library using a cutoff of 70% identity [11].

For multivariate statistical analysis, the XCMS output was further processed using Microsoft Excel (Microsoft, Redmond, WA, USA). Finally, the normalized data were imported into Simca-P software (version 13.0, http://www.umetrics.com/simca) for multivariate statistical analyses, including principal component analysis (PCA) and partial least-squares discriminant analysis (PLS–DA). All data were mean-centered and unit variance (UV)-scaled before PCA and PLS–DA applied to avoid overfitting. In the present study, a default 7-fold (Leave-1/7th samples-Out) cross validation procedure and 100 random permutations tests were performed to avoid overfitting of the supervised PLS-DA models.

Hierarchical cluster analysis (HCA) was performed and visualized by the “pheatmap” package available in R software (www.r-project.org). Pearson’s product-moment correlation was performed to calculate the correlation. Corresponding P-values and false discovery rate (FDR) of each correlation were also calculated using “cor.test function” [12] in R software. Identified metabolites were mapped onto general biochemical pathways according to annotation in Kyoto Encyclopedia of Genes and Genomes (KEGG) [13].

## Results

### Mammary gland tissue metabolic profiles

**Fig 1:**
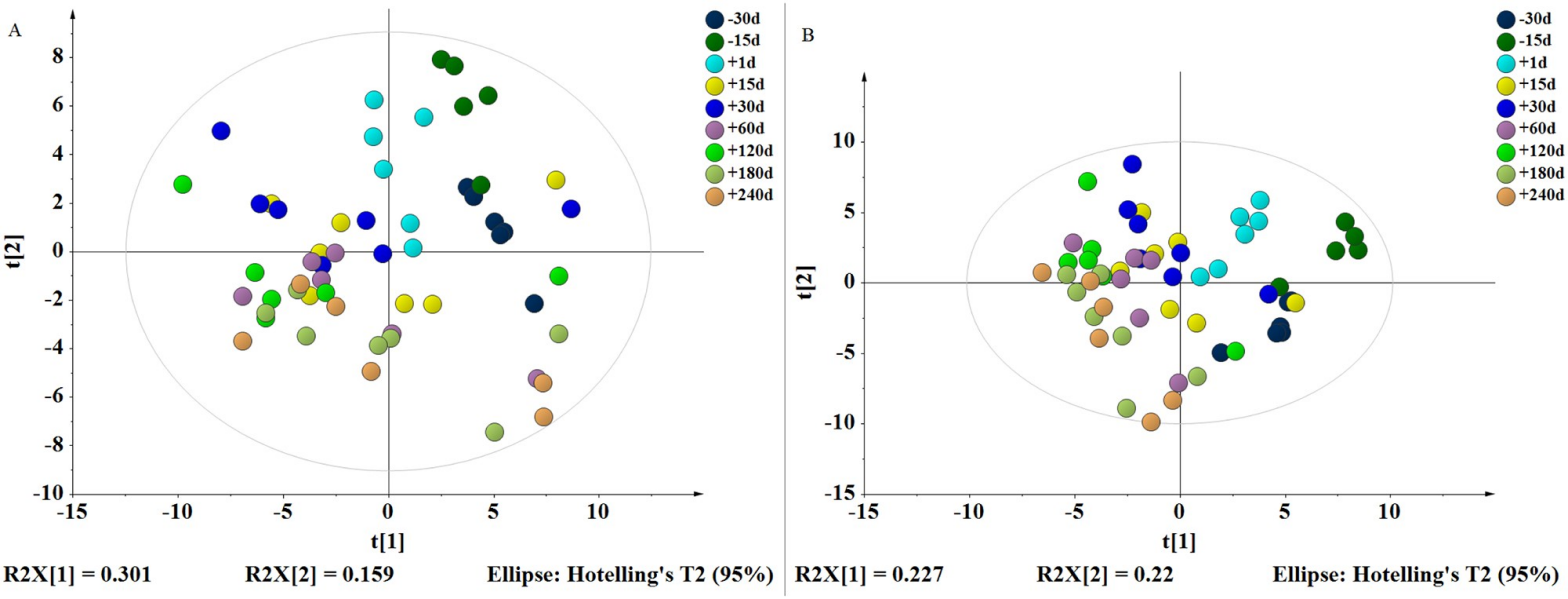
Differential metabolic profile in mammary tissue among the nine classes (-30d, -15d, +1d, +15d, +30d, +60d, +120d, +180d, +240d) and during the time courses of lactation cycle (pregnancy period, lactation period, dry period). (A) 2D PCA score plot depicting time-dependent trajectory of metabolic profile during the lactation cycle. (B) PLS-DA score plot separating nine classes’ samples.

**Fig 2:**
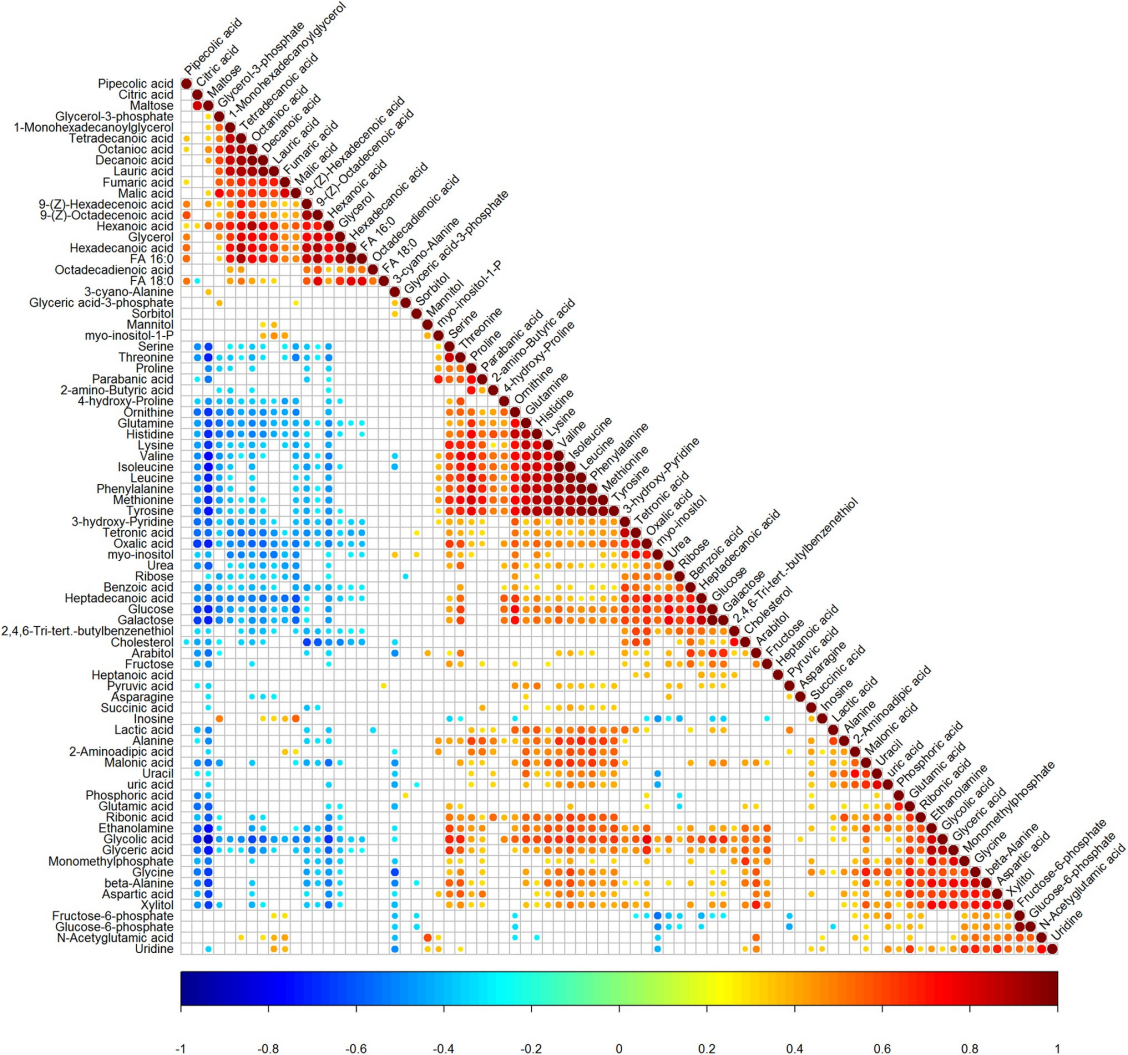
The ‘Pearson’ correlation analysis of 80 metabolites. (Red represents positive correlation, blue represents negative correlation. The blank portion represents P > 0.05, the colored portion represents P < 0.05).

In total, 80 retention time−exact mass pairs remained in each sample profile using the GC-MS analysis protocol and subsequent processes. As shown in S1 Fig. no drift was observed in the PCA scores plot representation of QC samples. Thus, the metabolic features demonstrated acceptable reproducibility and stability in GC−MS profiling analyses. Changes in the metabolite profile over the lactation cycle were observed. In addition, a plot of scores at different time phases on the axis of the first principal component (PC1, *R*^*2*^*X*_*1*_ = 0.30) accounted for a sizable portion of variance during the pregnancy, lactation and dry periods (Fig 1A). Subsequently, the PLS-DA models were established and demonstrated satisfactory modeling (*R*^*2*^*X* = 0.45, *R*^*2*^*Y* = 0.15, *Q*^*2*^ = 0.09), which showed differences of the nine classes during lactation in the direction of the first and second components (Fig 1B). The correlation of 80 metabolites was calculated by “Pearson” correlation coefficient, and the results are showed in Fig 2.

### Metabolic variations during different periods

**Fig 3:**
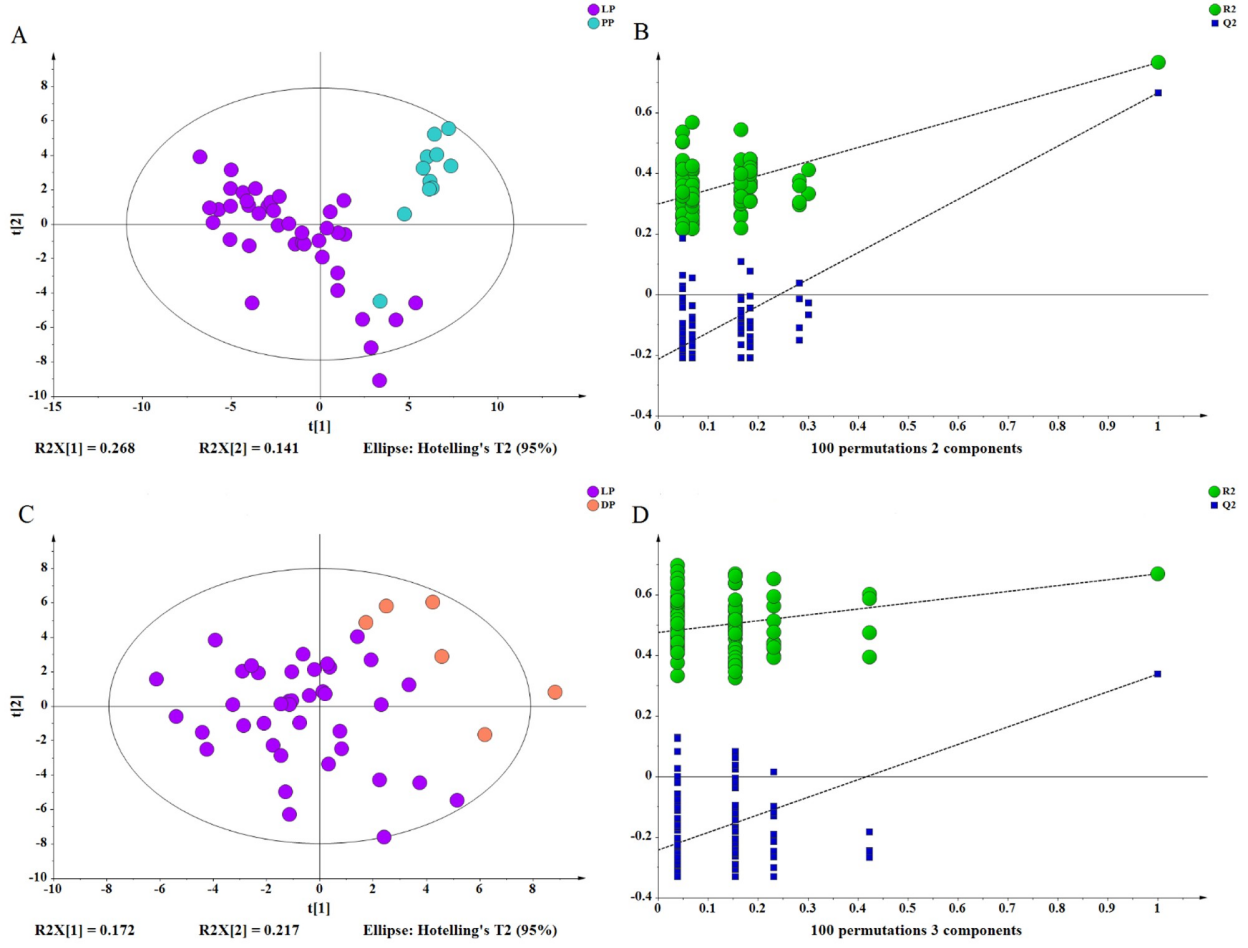
PLS-DA scores plot and permutation test. (A) PLS-DA scores plot discriminating pregnancy period (PP) from lactation period (LP), (B) Permutation test for the PLS-DA model A. (C) PLS-DA scores plot discriminating lactation period (LP) from dry period (DP), (D) Permutation test for the PLS-DA model C.

Based on the mammary gland tissue metabolic profiles, the scores plots and permutation tests of PLS-DA model discriminating the pregnancy period from the lactation period are presented in Fig 3, the results show a clear separation between the two groups. Mammary gland tissue metabolites passing the VIP threshold (VIP > 2) in this model (*R*^*2*^*X* = 0.41, *R*^*2*^*Y* = 0.77, *Q*^*2*^ = 0.67) were selected as significantly different between the pregnancy and lactation periods (Fig 3A and 3B). Similarly, another two-component PLS-DA model (*R*^*2*^*X* = 0.51, *R*^*2*^*Y* = 0.69, *Q*^*2*^ = 0.34) was also constructed to discriminate the dry period from the lactation period, achieving a clear discrimination between the two groups (Fig 3C and 3D). Although the *Q*^*2*^ value between the dry period and lactation period was slightly lower, this value was acceptable, considering the many uncontrollable factors. Model cross-validation through permutation tests (100 times) generated intercepts of *R*^*2*^ and *Q*^*2*^ (0.30 and -0.21 for pregnancy and lactation period, 0.48 and -0.24 for dry and lactation period, respectively). The low values of the intercepts indicate that the model was not over-fitted.

**Fig 4:**
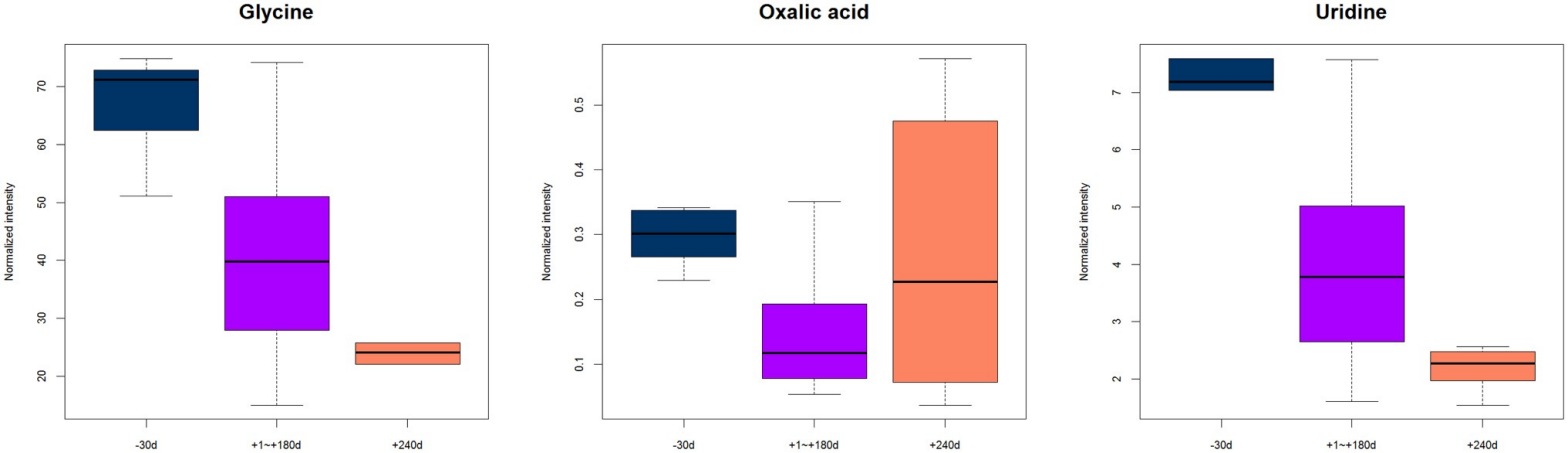
Box plots of differential metabolites throughout lactation cycle. Horizontal line in the middle portion of the boxes represents the median, bottom and top boundaries of boxes represent the lower and upper quartiles, respectively.

A list of 29 differential expressed metabolites (Table 1) including beta-alanine, aspartic acid, fructose, xylitol, arabitol, glycine and myo-inositol were identified by commercially available reference standards with MS/MS fragments and retention time (*P* < 0.05). As shown in Table 1, the relative concentration of 19 metabolites were significantly lower while 5 were significantly higher in the lactation period compared to pregnancy period. The relative concentration of 4 metabolites were significantly lower and 4 were significantly higher in the dry period compared to lactation period. These metabolites were carbohydrates, amino acids, lipids and their metabolites, suggesting that these metabolic pathways were altered through the lactation cycle. Compared to the lactation period, oxalic acid concentrations were higher during both pregnancy (*P* < 0.001, VIP = 1.17, fold-change = 0.51) and dry periods (*P* < 0.05, VIP = 1.59, fold-change = 1.78) (Fig 4). Glycine and uridine showed decreased levels during both the lactation and dry periods of the lactation cycle (lactation period VS. pregnancy period, P < 0.01, fold change: 0.59, 0.60, respectively; dry period VS lactation period, P < 0.05, fold change: 0.60, 0.54, respectively) (Fig 4).

**Table 1:** List of mammary gland tissue differential metabolites throughout lactation cycle.

NO.	Metabolites		LP vs PP ^a^			DP vs LP ^a^	
		*P* value ^b^	FC ^c^	VIP ^d^	*P* value ^b^	FC ^c^	VIP ^d^
1	Glyceric acid	9.04E-08	0.42	1.92			
2	beta-Alanine ^e^	4.26E-07	0.41	2.14			
3	Aspartic acid ^e^	1.03E-06	0.55	2.16			
4	Monomethylphosphate	1.66E-06	0.39	2.08			
5	Fructose ^e^	5.69E-06	0.42	1.95			
6	Glycolic acid	6.21E-06	0.56	1.70			
7	Xylitol ^e^	8.74E-06	0.56	1.85			
8	Maltose	1.87E-05	91.59	1.87			
9	Arabitol ^e^	6.86E-05	0.63	1.29			
10	Glycine ^e^	2.30E-04	0.59	1.93	2.22E-02	0.60	1.64
11	Ethanolamine	5.13E-04	0.54	1.77			
12	Oxalic acid	5.65E-04	0.51	1.17	2.68E-02	1.78	1.59
13	Uridine	2.15E-03	0.60	1.75	1.47E-02	0.54	1.74
14	Citric acid	2.90E-03	3.43	1.54			
15	Urea	4.19E-03	0.56	1.14			
16	Galactose	5.43E-03	0.59	1.06			
17	Threonine	6.03E-03	0.65	1.37			
18	Glucose	8.72E-03	0.61	0.99			
19	Asparagine	9.44E-03	0.62	0.50			
20	Octanioc acid	1.67E-02	3.54	1.07			
21	N-Acetyglutamic acid	2.26E-02	0.61	1.45			
22	Ribonic acid	2.96E-02	0.65	1.54			
23	Hexanoic acid	2.17E-02	2.61	1.16			
24	Decanoic acid	2.92E-02	3.07	0.77			
25	3-cyano-Alanine				9.44E-10	4.07	3.68
26	myo-inositol ^e^				4.22E-04	1.84	2.42
27	Sorbitol				3.32E-02	2.40	1.53
28	Fructose-6-phosphate				1.34E-02	0.66	1.76
29	Lauric acid				4.84E-02	0.49	1.42

^*a*^ PP, LP and DP denote pregnancy period, lactation period and dry period, respectively.

^*b*^ The *P* value calculated from two-tailed student’s t-test.

^*c*^ Fold change in the metabolite concentration.

^*d*^ Variable importance for the projection.

^*e*^ Metabolites are verified by reference standards.

### Metabolic KEGG pathway analysis

**Fig 5:**
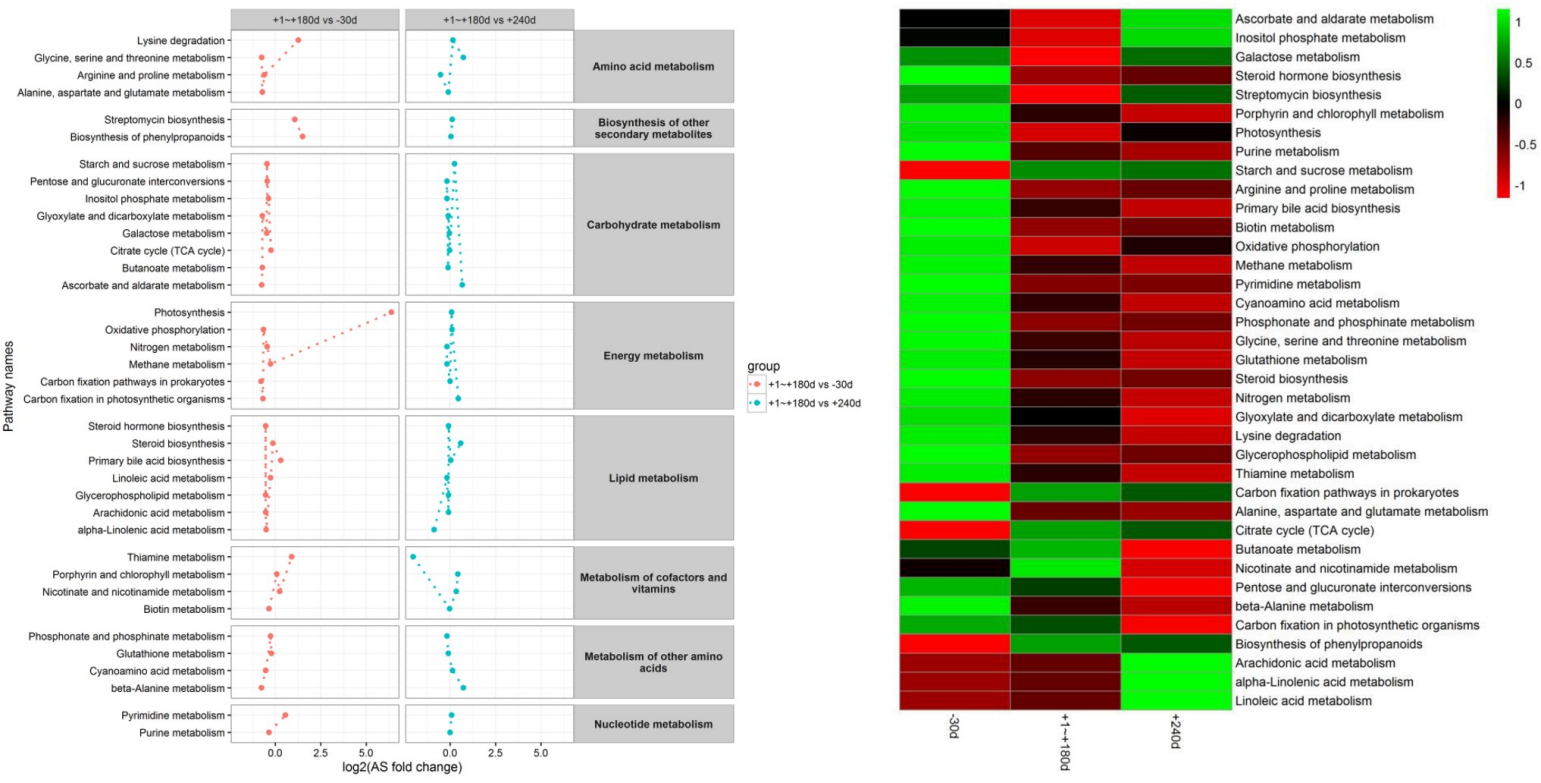
Variations and heatmap of metabolic pathways for 29 differential metabolites during the lactation cycle. (A) Variations of metabolic pathways during the lactation cycle, (B) Heatmap of metabolic pathways during the lactation cycle. (The bar color density represents the difference).

Overall, 37 pathways were obtained when the 29 differential metabolites were imported into the KEGG analysis. Fig 5 shows the functional enrichment of the different pathways. The most enriched functional pathways between lactation period and pregnancy period included glycine, serine and threonine metabolism (FDR < 0.001); alanine, aspartate and glutamate metabolism (FDR < 0.001); and TCA cycle (FDR = 0.008). The most enriched functional pathways between lactation and dry periods were glycine, serine and threonine metabolism (FDR < 0.001); glyoxylate and dicarboxylate metabolism (FDR = 0.01); and pyrimidine metabolism (FDR = 0.02). Among the 29 metabolites affected by the treatments, 5 are involved in the glycine, serine and threonine metabolism; 6 in the alanine, aspartate and glutamate metabolism; 4 in the TCA cycle; 9 in the glyoxylate and dicarboxylate metabolism; and 6 in the pyrimidine metabolism.

## Discussion

The defining characteristic of the mammalians is the provision of milk. Milk composition differs greatly across species, although species in the same taxonomic order tend to produce milk with a somewhat similar composition. The mammary gland is one of the few tissues in the body that undergoes repeated cycles of structural development, functional differentiation, and metabolism. However, these changes, particularly those related to the metabolism of the mammary gland, are still relatively unexplored. The inherent metabolic status during the lactation cycle play a vital role in the lactation physiology, which determines the milk production, udder health and dairy sustainability [14]. To our knowledge, this is the first paper addressing the lactation-related metabolic profiles using mammary gland tissue of yaks. This data provides novel insights and a greater understanding of the metabolic mechanism during the lactation cycle of yaks, and can guide further research to improve milk yield and enhance the constituents.

As shown in Fig 3A and 3B, PLS-DA scores plot revealed a clear and statistically significant separation of the lactation period versus the pregnancy period and the dry period versus the lactation period. A total of 29 differential metabolites were identified throughout the lactation cycle, and these differential metabolites were involved in pathways including amino acid, biosynthesis of other secondary metabolites, carbohydrate, energy, lipid, cofactors and vitamins and nucleotide metabolism, indicating that these metabolic pathways were influenced by different periods of the lactation cycle.

The significant changes observed in mammary gland tissue composition throughout lactation cycle are primarily related to the identified compounds: oxalic acid, glycine, and uridine. Oxalic acid has usually been seen as an inert end product of the metabolism in animals because mammalian cells cannot metabolize it [15]. However, recent studies indicate that the levels of oxalate are too high for the substance to only be an end-product of the metabolism in animals [16]. Therefore, it has been suggested that there could be an oxalate oxidase pathway in animals which uses oxalate to promote phagocytes [17]. Oxamide is a diamide that is derived from oxalic acid and participates in the TCA cycle, and the TCA cycle is one of the most important pathways in the mammary grand for lactation initiation [18, 19]. The expression of oxalic acid was showing significantly lower in the mammary grand tissues of lactating than non-lactating cows [18] which is consistent with ours. Citric acid (fold-change = 5.50) and other secondary metabolites participating in the TCA cycle were significantly higher in the lactating yaks, indicating that the TCA cycle is more active during lactation initiation. The changes of the expression of oxalic acid was presumed to be related to the up-regulated TCA cycle.

Glycine is the simplest amino acid and a basic nutrient. As part of endogenous antioxidant glutathione, glycine is a semiessential amino acid. Glycine is primarily involved in the proliferation of immune cells and synthesis of proteins related to inflammatory and immune responses [20–23]. Additionally, glycine can also regulate the proliferation and apoptosis of mammary epithelial cells of dairy cows and promote the conversion of mammary epithelial cells to the S phase and cell division [24]. Two studies have reported that glycine was the most abundant metabolite in the goat mammary gland [25] and lactating dairy cow mammary grand [18]. Glycine metabolism pathway is important in the mammary gland for lactation initiation, because glycine can be used to synthesize creatine with arginine and methionine. It is reported that more creatine is needed to ease the serious negative energy balance in dairy cows [26]. We speculate that the decreased level of glycine was related to the negative energy balance in yaks.

Uridine is one of the five standard nucleosides that make up nucleic acids. The relative concentration of uridine was significantly lower in Holstein than yak and other dairy animals [7]. Besides, the level of uridine was identified significantly higher in the lactation than non-lactation cows and that was consistent with our result [18]. Uridine metabolism can be regulated by the prolactin stimulation, the uridine uptake and its incorporation in to RNA are concomitantly stimulated by prolactin in mammary gland [27]. In mammals, prolactin stimulates the mammary gland to produce milk and has important cell cycle-related functions such as a growth factor and differentiating factor [28]. The prolactin levels increase concentration during pregnancy cause enlargement of the mammary glands and preparing for milk production and fall when the process of breastfeeding is terminated. Therefore, we speculate that the decreased level of uridine was affected by the decreased prolactin level.

In our study, all of the shared metabolites in the mammary grand from the pregnancy, lactation and dry periods were evaluated together. For the lactation yaks, the most active and important pathways included glycine, serine and threonine metabolism, alanine, aspartate and glutamate metabolism, TCA cycle, glyoxylate and dicarboxylate metabolism and pyrimidine metabolism. Among them, TCA cycle is the key pathways of carbohydrate metabolism and plays a central role in cellular respiration and the supply of energy to all living cells [29], which is of paramount significance to the cell’s metabolic efficiency and, therefore, to the yak’s metabolism and production. The differential expression level of citric acid between the lactating and non-lactating cows (fold-change = 5.50) [18] was consistent with our results (fold-change = 3.43). Citric acid is formed in the TCA cycle or from the diet and participates in the intermediate metabolism of carbohydrate oxidation in animal tissues [30]. The level of citric acid is significantly higher in the lactating yaks and may enhance energy by participating in the TCA cycle. TCA cycle and glycine, serine and threonine metabolism pathways are the most important and work together in the mammary grand for lactation initiation [18, 31]. As a prime metabolic source of glutathione, creatine, purines and serine and a protein constituent in the lactating mammary epithelial cells, glycine plays a significant role in various biological processes such as ischemia reperfusion injury, oxygen stress, cell membrane injury, tumor metastasis and so on [32, 33]. Glyoxylate and dicarboxylate metabolism pathway were determined to be pathways that were significantly impacted in the lactation cows [12]. In the alanine, aspartate and glutamate metabolism pathway, aspartate is a precursor of many compounds that are involved in cellular signaling, such as N-acetyl-aspartate. It is also a metabolite in the urea cycle and participates in gluconeogenesis in dairy cows [34]. Pyrimidines are involved in growth via DNA and RNA formation and they also have a special role in mammary grand because of the essential role of uridine in the synthesis of lactose and other uridine-sugar complexes, and the vital role of cytidine in the synthesis of complex lipids resulting from the mechanism of milk secretion [35]. Thymine is broken down into β-aminoisobutyrate which can be further broken down into intermediates eventually leading into the TCA cycle. Based on the overview map of the KEGG pathway, these 5 key metabolic pathways were closely integrated together, suggesting that the carbohydrate pathways, amino acid pathways and nucleotide pathway play vital roles in regulating lactation maintenance for the yak.

## Conclusion

The present study investigates the metabolite profile of yak mammary gland tissue during pregnancy, lactation and dry periods by gas chromatography/mass spectrometry. The analysis revealed substantial metabolic alteration through the lactation cycle; 29 metabolites were up or downregulated during the lactation cycle. Thirty-seven pathways were obtained when the 29 differential metabolites were imported into the KEGG analysis; the main pathways affected by the lactation cycle were glycine, serine and threonine metabolism; aspartate and glutamate metabolism; TCA cycle; glyoxylate and dicarboxylate metabolism; and pyrimidine metabolism. Overall, our results provide a better physiological understanding of the lactation metabolism of yaks, which can help elucidate the regulated metabolic strategies for the lactation yaks in the future.

## Supporting information

S1 Fig 2D PCA scores plot for consecutively analyzed QC samples (Red indicates QC samples). (TIF)

## References

[1] Neupaney D, Kim JB, Ishioroshi M, Samejima K. Study on some functional and compositional properties of yak butter lipid. Anim Sci J. 2003; 74(5): 391–397. 10.1046/j.1344-3941.2003.00131.x.

[2] Dong SK, Long RJ, Kang MY. Milking performance of China yak (Bos grunniens): A preliminary report. Afr J Agr Res. 2007; 2(3): 52–57.

[3] Jiang M, Lee JN, Bionaz M, Deng XY, Wang Y. Evaluation of Suitable Internal Control Genes for RT-qPCR in Yak Mammary gland tissue during the Lactation Cycle. PLoS One. 2016; 11(1): e0147705 10.1371/journal.pone.0147705. 26808329 PMC4726593

[4] Zhong JC, Zi XD, Han JL, Chen XH. Yak production in central Asian highlands. Proceedings of the fourth international congress on yak. 2004; 32–36.

[5] Nikkhah A. Science of Camel and Yak Milks: Human Nutrition and Health Perspectives. Food Nutr Sci. 2011; 2(6): 667–673. 10.4236/fns.2011.26092.

[6] Loor JJ, Bionaz M, Drackley JK. Systems physiology in dairy cattle: nutritional genomics and beyond. Annu Rev Anim Biosci. 2013; 1: 365–392. 10.1146/annurev-animal-031412-103728 . 25387024

[7] Yang Y, Zheng N, Zhao X, Zhang Y, Han R, Yang J, et al Metabolomic biomarkers identify differences in milk produced by Holstein cows and other minor dairy animals. J Proteomics. 2016; 136: 174–182. 10.1016/j.jprot.2015.12.031 . 26779989

[8] Bionaz M, Loor JJ. Identification of reference genes for quantitative real-time PCR in the bovine mammary gland during the lactation cycle. Physiol Genomics. 2007; 29(3): 312–319. 10.1152/physiolgenomics.00223.2006 . 17284669

[9] Smith CA, Want EJ, O’Maille G, Abagyan R, Siuzdak G. XCMS: processing mass spectrometry data for metabolite profiling using nonlinear peak alignment, matching, and identification. Anal Chem. 2006; 78(3): 779–787, 10.1021/ac051437y . 16448051

[10] Vanholme R, Storme V, Vanholme B, Sundin L, Christensen JH, Goeminne G, et al A systems biology view of responses to lignin biosynthesis perturbations in Arabidopsis. Plant Cell. 2012; 24(9): 3506–3529. 10.1105/tpc.112.102574 . 23012438 PMC3480285

[11] Abu Dawud R, Schreiber K, Schomburg D, Adjaye J. Human embryonic stem cells and embryonal carcinoma cells have overlapping and distinct metabolic signatures. PLoS One. 2012; 7(6): e39896 10.1371/journal.pone.0039896 . 22768158 PMC3387229

[12] Benjamini Y, Yekutieli D. The control of the false discovery rate in multiple testing under dependency. Ann Stat. 2008; 29(4): 1165–1188. 10.1214/aos/1013699998.

[13] Kanehisa M, Goto S. KEGG: kyoto encyclopedia of genes and genomes. Nucleic Acids Res. 2000; 28(1): 27–30. 10.1093/nar/28.1.27 . 10592173 PMC102409

[14] Sejrsen K, Hvelplund Torben, Nielsen MO. Ruminant physiology: digestion, metabolism and impact of nutrition on gene expression, immunology and stress. The Netherlands: Wageningen Academic Publishers 2006 10.3920/978-90-8686-566-6

[15] Castellaro AM, Tonda A, Cejas HH, Ferreyra H, Caputto BL, Pucci OA, Gil GA. Oxalate induces breast cancer. BMC Cancer. 2015; 15: 761 10.1186/s12885-015-1747-2 . 26493452 PMC4618885

[16] Allison MJ, Littledike ET, James LF. Changes in ruminal oxalate degradation rates associated with adaptation to oxalate ingestion. J Anim Sci. 1977;45(5): 1173–1179. 10.2527/jas1977.4551173x . 599103

[17] ÇalişKan M. The Metabolism of Oxalic Acid. Turk J Zool. 1998; 24(2000): 103–106.

[18] Sun HZ, Shi K, Wu XH, Xue MY, Wei ZH, Liu JX, Liu HY. Lactation-related metabolic mechanism investigated based on mammary gland metabolomics and 4 biofluids’ metabolomics relationships in dairy cows. BMC Genomics. 2017; 18(1): 936 10.1186/s12864-017-4314-1 . 29197344 PMC5712200

[19] Antunes-Fernandes EC, van Gastelen S, Dijkstra J, Hettinga KA, Vervoort J. Milk metabolome relates enteric methane emission to milk synthesis and energy metabolism pathways. J Dairy Sci. 2016; 99(8): 6251–6262. 10.3168/jds.2015-10248 . 27236769

[20] Alarcon-Aguilar FJ, Almanza-Perez J, Blancas G, Angeles S, Garcia-Macedo R, Roman R, Cruz M. Glycine regulates the production of pro-inflammatory cytokines in lean and monosodium glutamate-obese mice. Eur J Pharmacol. 2008; 599(1–3): 152–158. 10.1016/j.ejphar.2008.09.047 . 18930730

[21] Garcia-Macedo R, Sanchez-Muñoz F, Almanza-Perez JC, Duran-Reyes G, Alarcon-Aguilar F, Cruz M. Glycine increases mRNA adiponectin and diminishes pro-inflammatory adipokines expression in 3T3-L1 cells. Eur J Pharmacol. 2008; 587(1–3): 317–321. 10.1016/j.ejphar.2008.03.051 . 18499099

[22] Thelwall PE, Simpson NE, Rabbani ZN, Clark MD, Pourdeyhimi R, Macdonald JM. In vivo MR studies of glycine and glutathione metabolism in a rat mammary tumor. NMR Biomed. 2012; 25(2): 271–278. 10.1002/nbm.1745 . 21751272 PMC3193887

[23] Wheeler MD, Ikejema K, Enomoto N, Stacklewitz RF, Seabra V, Zhong Z, et al Glycine: a new anti-inflammatory immunonutrient. Cell Mol Life Sci. 1999; 56(9–10): 843–856. 10.1007/s000180050030 . 11212343 PMC11147092

[24] Tang MH, Zhang XF, Dan N, Ao CJ, Gao M. Effects of Glycine on Proliferation and Apoptosis of Mammary Epithelial Cells of Dairy Cows. J Anim Nutr. 2014; 26(8): 2162–2168.

[25] Palma M, Hernández-Castellano LE, Castro N, Arguëllo A, Capote J, Matzapetakis M, de Almeida AM. NMR-metabolomics profiling of mammary gland secretory tissue and milk serum in two goat breeds with different levels of tolerance to seasonal weight loss. Mol Biosyst. 2016; 12(7): 2094–2107. 10.1039/c5mb00851d . 27001028

[26] Wang YH, Gao Y, Xia C, Zhang HY, Qian WD, Cao Y. Pathway analysis of plasma different metabolites for dairy cow ketosis. Ital J Anim Sci. 2016; 15(3): 545–551. https://www.tandfonline.com/doi/full/10.1080/1828051X.2016.1180643.

[27] Rillema JA. Characteristics of the prolactin stimulation of uridine metabolism in mammary gland explants. Endocrinology. 1975; 96(5): 1307–1311, 10.1210/endo-96-5-1307 . 1122890

[28] Oakes SR, Rogers RL, Naylor MJ, Ormandy CJ. Prolactin regulation of mammary gland development. J Mammary Gland Biol Neoplasia. 2008; 13(1): 13–28. 10.1007/s10911-008-9069-5 . 18219564

[29] Grassian AR, Parker SJ, Davidson SM, Divakaruni AS, Green CR, Zhang X. et al IDH1 mutations alter citric acid cycle metabolism and increase dependence on oxidative mitochondrial metabolism. Cancer Res. 2014; 74(12): 3317–3331. 10.1158/0008-5472.CAN-14-0772-T . 24755473 PMC4885639

[30] Krebs HA, Johnson WA. The role of citric acid in intermediate metabolism in animal tissues. FEBS Lett. 1980; 117(S1): 2–10. 10.4159/harvard.9780674366701.c143 6998725

[31] Sun HZ, Wang DM, Wang B, Wang JK, Liu HY, Guan le L, Liu JX. Metabolomics of four biofluids from dairy cows: potential biomarkers for milk production and quality. J Proteome Res. 2015; 14(2): 1287–1298. 10.1021/pr501305g . 25599412

[32] Shennan DB, McNeillie SA, Curran DE. The effect of a hyposmotic shock on amino acid efflux from lactating rat mammary tissue: stimulation of taurine and glycine efflux via a pathway distinct from anion exchange and volume-activated anion channels. Exp Physiol. 1994; 79(5): 797–808. . 7529510 10.1113/expphysiol.1994.sp003808

[33] Zhang WC, Shyh-Chang N, Yang H, Rai A, Umashankar S, Ma S. et al Glycine decarboxylase activity drives non-small cell lung cancer tumor-initiating cells and tumorigenesis. Cell. 2012; 148(1–2): 259–272. 10.1016/j.cell.2011.11.050 . 22225612

[34] Piccioli-Cappelli F, Loor JJ, Seal CJ, Minuti A, Trevisi E. Effect of dietary starch level and high rumen-undegradable protein on endocrine-metabolic status, milk yield, and milk composition in dairy cows during early and late lactation. J Dairy Sci. 2014; 97(12): 7788–7803. 10.3168/jds.2014-8336 . 25459908

[35] Kunjara S, Sochor M, Bennett M, Greenbaum AL, McLean P. Pyrimidine nucleotide synthesis in the rat mammary gland: changes in the lactation cycle and effects of diabetes. Biochem Med Metab Biol. 1992; 48(3):263–274. . 1476792 10.1016/0885-4505(92)90073-8

