# Supplementary figures and images for: Metabolomic profiles in yak mammary gland tissue during the lactation cycle
Source: PLoS One. 2019 Jul 5;14(7):e0219220. doi: 10.1371/journal.pone.0219220 (PMC6611666; doi:10.1371/journal.pone.0219220)

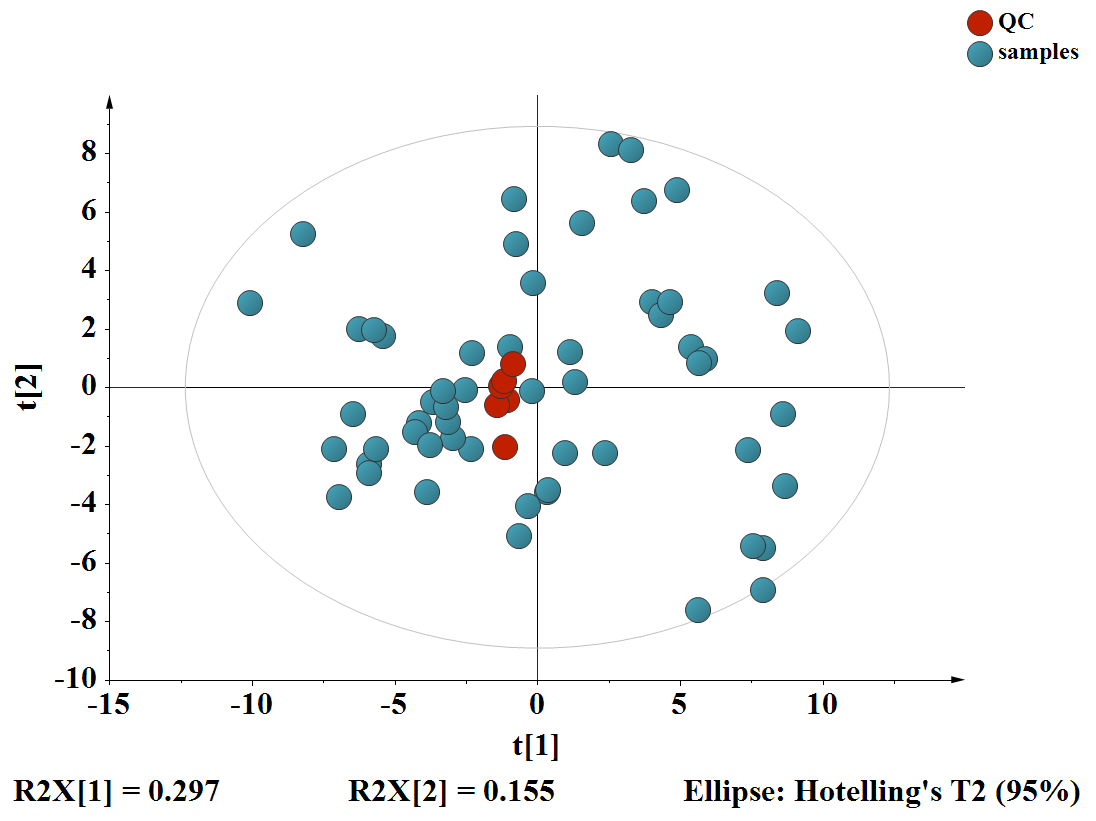

Supplement: S1 Fig — (TIF) [file pone.0219220.s001.tif]
